# Current Issues and Future Technologies in Esophageal Cancer Surgery

**DOI:** 10.3390/jcm12010209

**Published:** 2022-12-27

**Authors:** Christian Denecke, Johann Pratschke, Jonas Raakow

**Affiliations:** Chirurgische Klinik, Campus Charité Mitte|Campus Virchow-Klinikum, Charité Universitätsmedizin, 10117 Berlin, Germany

Gastrointestinal surgery has evolved rapidly in recent years, with laparoscopic techniques being implemented as the standard procedure and robotic surgery becoming increasingly important. While minimally invasive procedures allow for a significantly lower morbidity, higher lymph node yield, and faster recovery after surgery, numerous questions remain a matter of debate in esophageal and gastric cancer surgery. Thus, our current Special Issue focuses on current and future questions in upper gastrointestinal surgery to highlight some of the most urgent challenges and innovations in this field. While technical progress continuously impacts surgical techniques, anastomotic healing remains the Achilles heel of minimally invasive esophagectomy (MIE). Given substantial variations in anastomotic techniques and leakage rates ranging between 8% and 24% [1], Bartella et al. used a two-round Delphi process to highlight three main techniques for intrathoracic anastomoses conducted by experts: end-to-side circular stapling, end-to-side double stapling, and side-to side linear stapling [2]. While a recent meta-analysis described comparable leak rates following linear and circular stapled anastomoses [3], Schröder et al. published a well-recognized database analysis proving a significantly lower leak rate after end-to-side purse string anastomosis (13.9%) compared to side-to-side linear stapling (15.6%) [4]. In addition, several technical adjuncts, such as the use of an omental wrap, the diameter of the circular stapling device, intraoperative perfusion monitoring, the use of a preemptive endoluminal vacuum sponge, ischemic preconditioning, and many more contribute to the complexity of the gastroesophageal anastomotic technique. In regard to the size of the circular anastomosis, our group addressed this question in the Special Issue “Advances in minimally invasive gastrointestinal surgery” [5]. In a single-center analysis, a 29 mm circular anastomosis at the vena azygos level was associated with a significantly lower leak rate than a 25 mm circular anastomosis (3.7% vs. 18.5%). In contrast, a study from Mainz did not observe any impact of a larger circular diameter size on anastomotic complications, underlining that, despite all technical progress, anastomotic techniques require future evaluation, i.e., by randomized controlled trials [6].

As minimally invasive Upper GI Surgery remains technically challenging, it is our task and duty to train younger surgeons without compromising surgical quality and oncological outcome. In our current Special Issue, Seika et al. analyzed the learning curve for laparoscopic gastrectomy, which was completed after 44 cases [7]. Interestingly, performing the learning curve did not impact on anastomotic leakage rate, mortality, or overall survival, while surgical and oncological quality criteria were unaltered. As both teaching and quality assessment become increasingly important, these results seem promising with regards to both meeting high surgical and economic standards and simultaneously teaching young fellows.

Meanwhile, complication management and “failure to rescue” have gained more attention in recent years. While the use of endoscopic stents has been established in the treatment of anastomotic leaks for many years, nowadays endoscopic vacuum therapy (EVT, endosponge) is the new gold standard in many centers. A meta-analysis by Rausa et al. showed a superior success rate in anastomotic healing, shorter treatment duration, and a lower complication rate of EVT vs. endoscopic stent placement [8]. In contrast, Berlth et al. published comparable outcomes of Stent vs. Endosponge treatment [9]. Even though data are scarce on this matter, endosponge therapy is on the rise and its preemptive use in high-risk anastomoses or ischemic mucosa conditions has been published with good results [10]. Specifically, morbidity was lowered, and anastomotic leak rates were decreased to 5–7.5% when an endosponge was applied intraoperatively [10,11]. Given the demographic changes with an increasing proportion of elderly patients with a higher ASA score, this concept demonstrates the potential to further improve anastomotic leakage rates. However, the impact of preoperative radiochemotherapy on EVT success rates remains uncertain. Seika et al. presented a single center study in this Special Issue on EVT, with patients with an anastomotic leak after esophagectomy who underwent preoperative radiochemotherapy (nRCT) compared with patients who were subject to preoperative chemotherapy [12]. Not surprisingly, the duration of EVT was significantly longer for anastomotic leaks in patients after nRCT and leakage treatment often required multimodal treatment, but overall success rates did not differ. Thus, the potential of endovac therapy has been proven, leading to its preemptive use in high-risk situations and future developments, such as the recently introduced VACStent.

In addition to laparoscopic or robotic techniques, innovations and further developments in intraoperative real-time imaging have recently gained significant importance in esophageal surgery. The goal is to use invasive or noninvasive procedures to optimize the surgical process, reduce postoperative complications, and thus increase patient safety. Currently, the most promising techniques are fluorescence imaging with indocyanine green (ICG), which is already widely used clinically, and hyperspectral imaging (HSI). Current questions are mainly related to the extension of the clinical application of ICG imaging and the application of HSI for non-invasive perfusion analysis and tissue differentiation. In principle, ICG imaging is well suited for a categorical assessment (yes vs. no) regarding anatomic structures or perfusion. However, it is not well suited for graduated assessments, such as the level of perfusion or the differentiation of arterial ischemia and venous congestion. One of the major advantages of ICG-based perfusion analysis seems to be the determination of the ideal anastomosis position at the gastric conduit in the context of oncological esophagectomy. A recent meta-analysis by Van Daele et al. demonstrated a significantly reduced rate of anastomotic leaks of esophago-gastric anastomoses after assessing the gastric tube perfusion with ICG (10% in ICG-guided vs. 20.6% in control group; *p* < 0.001) [13]. The authors concluded that the lower anastomotic leak rate in the well-perfused group suggest that a good fluorescent signal predicts a good outcome. Using another meta-analysis, Slooter et al. reported an adjustment of the surgical approach in approximately one quarter of cases with the use of ICG [14]. Kumagai et al. described the “90-second rule” to verify good perfusion of the anastomotic region on the reconstructed gastric tube [15]. The anastomoses in 70 patients were created in regions in which less than 90 s was required from the appearance of the fluorescence signal at the base of the arcade to the accumulation of the dye in the corresponding area. Other uses of ICG imaging include lymph node mapping or localization of metastases, as well as imaging of the thoracic duct to prevent chyle leaks during esophagectomy. These are all promising results, but there is still no standardized application protocol, especially regarding the required ICG concentration and the required observation time after the application. HIS, on the other hand, is a new method of “image-guided precision surgery” that has already yielded promising results in quantitative perfusion analysis and tissue and tumor detection and characterization. HIS is currently in an experimental phase and few clinical data are available for esophageal resection [16]. To date, one of the main limitations is that the required camera is only available for open surgery. A minimally invasive version of the camera has been developed and can be used experimentally ex situ, but it is not yet approved for use in humans [17]. The subject of current investigations is whether the spectroscopic properties analyzed by means of HSI also allow for reliable and clinically valuable tissue differentiation and tumor detection, and whether they can be automatically evaluated in the future using machine learning and artificial intelligence.
